# Topical Ocular TRPV1 Antagonist SAF312 (Libvatrep) Demonstrates Safety, Low Systemic Exposure, and No Anesthetic Effect in Healthy Participants

**DOI:** 10.1167/tvst.11.11.15

**Published:** 2022-11-17

**Authors:** Kalliopi Stasi, Qusai Alshare, Monish Jain, Michael Wald, Yifang Li

**Affiliations:** 1 Novartis Institutes for BioMedical Research, Cambridge, MA, USA

**Keywords:** SAF312, Libvatrep, TRPV1, ocular surface pain, cornea, esthesiometry

## Abstract

**Purpose:**

This first-in-human (FIH) study evaluated the safety, tolerability, pharmacokinetics, and effect on corneal sensitivity of topical ocular SAF312 in healthy participants.

**Methods:**

This double-masked, randomized study comprised single-ascending dose (SAD), multiple-ascending dose (MAD), and esthesiometry parts. In SAD and MAD, 8 participants in each dose cohort were randomized 3:1 to receive SAF312 or vehicle, 1 drop once (SAD), or 1 drop 4 or 8 times daily for 7 days (MAD). Safety and pharmacokinetics were the primary and secondary objectives. Blink rate, tear production, tear film break-up time (TFBUT), and corneal sensitivity were also explored.

**Results:**

SAF312 was tolerated in single and multiple doses, including the maximum concentration of 2.5% dosed up to 1 drop 8 times daily for 7 days. Most adverse events (AEs) were mild and similar between SAF312 and vehicle-treated groups. No serious AEs were reported. SAF312 was rapidly absorbed, and had low systemic exposure. After supratherapeutic dosing for 7 days, mean steady-state exposures of SAF312 were low and afforded safety margins of >70-fold compared with no-observed-AE levels following oral dosing in preclinical studies. No clinically relevant changes were observed in blink rate, tear production, and TFBUT. SAF312 showed no undesired anesthetic effect on the cornea.

**Conclusions:**

SAF312 was well tolerated, with no ocular or systemic safety concerns; had no anesthetic effect, and demonstrated rapid topical absorption with low systemic exposure.

**Translational Relevance:**

This work bridges the gap between basic research and clinical care by providing FIH data of SAF312, supporting the further investigation as a potential treatment for ocular surface pain.

## Introduction

The human cornea has the highest sensory innervation in the body with a dense subepithelial nerve plexus and has the potential to be a powerful generator of pain.^1^ Ocular surface pain (OSP), whether acute or chronic, may arise due to various causes, such as infection, inflammation, or trauma at the ocular surface or changes to the peripheral or central nerves in the ocular surface sensory pathway.^2^ OSP remains an ill-defined entity and can be perceived as pain, discomfort, aching, burning, irritation, dryness, and grittiness.^2^^,^^3^ OSP is often present within the scope of other ocular conditions, such as dry eye disease (DED),^2^^,^^4^^,^^5^ accounting for approximately 30% prevalence in the general population and, placing a huge treatment burden on healthcare systems.^2^ Moreover, OSP has a negative impact on the quality of life of affected individuals, making it an important physical and mental health concern.^2^^,^^6^

The treatment of OSP has been identified as one of the highest unmet needs in patients with ocular surface diseases as currently neither an established standard of care nor topical treatments for long-term use currently exist.^7^^,^^8^ The use of currently available analgesics, such as oral pain medications (acetaminophen with codeine/opiates), are constrained by their gradual onset and limited efficacy; the undesirable or adverse side effects of systemic opioids are well known.^8^^–^^12^ Further, continuous topical application of local anesthetics or topical nonsteroidal anti-inflammatory drugs (NSAIDs) is known to increase the incidence of infections and corneal scarring and delay corneal wound healing in some patients with epithelial defects; long-term use can increase the risk of severe corneal events, such as corneal melts.^13^^–^^16^

The transient receptor potential vanilloid subtype 1 (TRPV1) receptor is a nociceptor that maintains a dual function in corneal tissues by participating in pain sensing, transmission, and regulation and acting as a mediator of innate inflammatory responses (Stasi K., et al. IOVS 2021;62: ARVO E-Abstract 3538424).^8^^,^^17^^–^^19^ TRPV1 channel inhibitors aim to prevent pain by blocking a receptor where pain is generated; this action makes them ideal candidates for treating ocular pain.^8^^,^^20^^,^^21^ Recently, SYL1001, a small interfering RNA (siRNA) developed for the specific inhibition of TRPV1 in DED, showed a well-tolerated safety profile and the ability to reduce hyperemia and ocular pain or discomfort in phase I, II, and III studies.^8^^,^^22^^–^^24^ Although multiple selective orally administered TRPV1 antagonist molecules have been explored for over a decade, their development in clinical use was impeded by systemic liabilities, such as hyperthermia or a decrease in noxious heat detection.^8^^,^^25^^–^^28^ Furthermore, to the best of our knowledge, with the notable exception of the silencing RNA SYL1001,^8^ no known low-molecular-weight TRPV1 inhibitor has ever been clinically tested for topical ocular use.

SAF312 (Libvatrep) is a potent, highly selective, noncompetitive inhibitor of TRPV1 (Medley Q. Y. J., et al. IOVS 2021;62: ARVO E-Abstract 3532343), a key mediator of ocular pain that is expressed in the corneal and conjunctival tissues (Stasi K., et al. IOVS 2021;62: ARVO E-Abstract 3538424 and Medley Q. Y. J., et al. IOVS 2021;62: ARVO E-Abstract 3532343).^29^ SAF312 was initially developed as an oral formulation and evaluated in clinical trials for managing postoperative dental pain.^30^ Oral SAF312 showed efficacy in reducing dental pain; however, the systemic side effect of heat insensitivity (monitored with a hand immersion test in warm water) precluded its further clinical development.^8^^,^^30^ Similarly, in another study, systemic administration of the TRPV-1 antagonist AMG517 induced marked and persistent hyperthermia (up to 40.2°C), which resulted in the termination of a phase Ib dental pain study.^28^

Topical ocular formulation typically can provide good topical exposure with minimal systemic absorption; therefore, this route of administration has the theoretical advantage of providing topical efficacy while avoiding systemic adverse events (AEs) of oral or systemic routes of administration.^31^ Topical ocular SAF312 in a preclinical pharmacokinetic (PK) analysis in rabbit ocular tissues and plasma showed the highest exposure in the cornea and conjunctiva, followed by the aqueous humor, lens, retina, plasma, and vitreous humor (Medley Q. Y. J., et al. IOVS 2021;62: ARVO E-Abstract 3532343). Low systemic exposure suggested reduced risk of systemic side effects. *In vivo* toxicology studies in a rabbit model showed no clinical or histopathological findings following topical ocular doses of up to 2.5% administered 8 times daily per eye (approximately 7.4 mg/d human equivalent). Additionally, no delay was observed in corneal wound healing after topical SAF312 administration up to a dose of 2.5% 4 times daily (3.7 mg/d) for 14 days in rabbits after photorefractive keratectomy (PRK; Stasi K., et al. IOVS 2021;62: ARVO E-Abstract 3538424).

These findings from preclinical studies support the clinical development of topical ocular SAF312 as a potential first-in-class topical ocular analgesic for the treatment of OSP. The objectives of this first-in-human (FIH) study were to assess the safety, tolerability, and PKs of single and multiple ascending doses of SAF312 as topical ocular drops in adult healthy participants and explore the maximum tolerated dose (MTD) and its effect on corneal sensitivity.

## Methods

### Study Design

**Figure 1.**
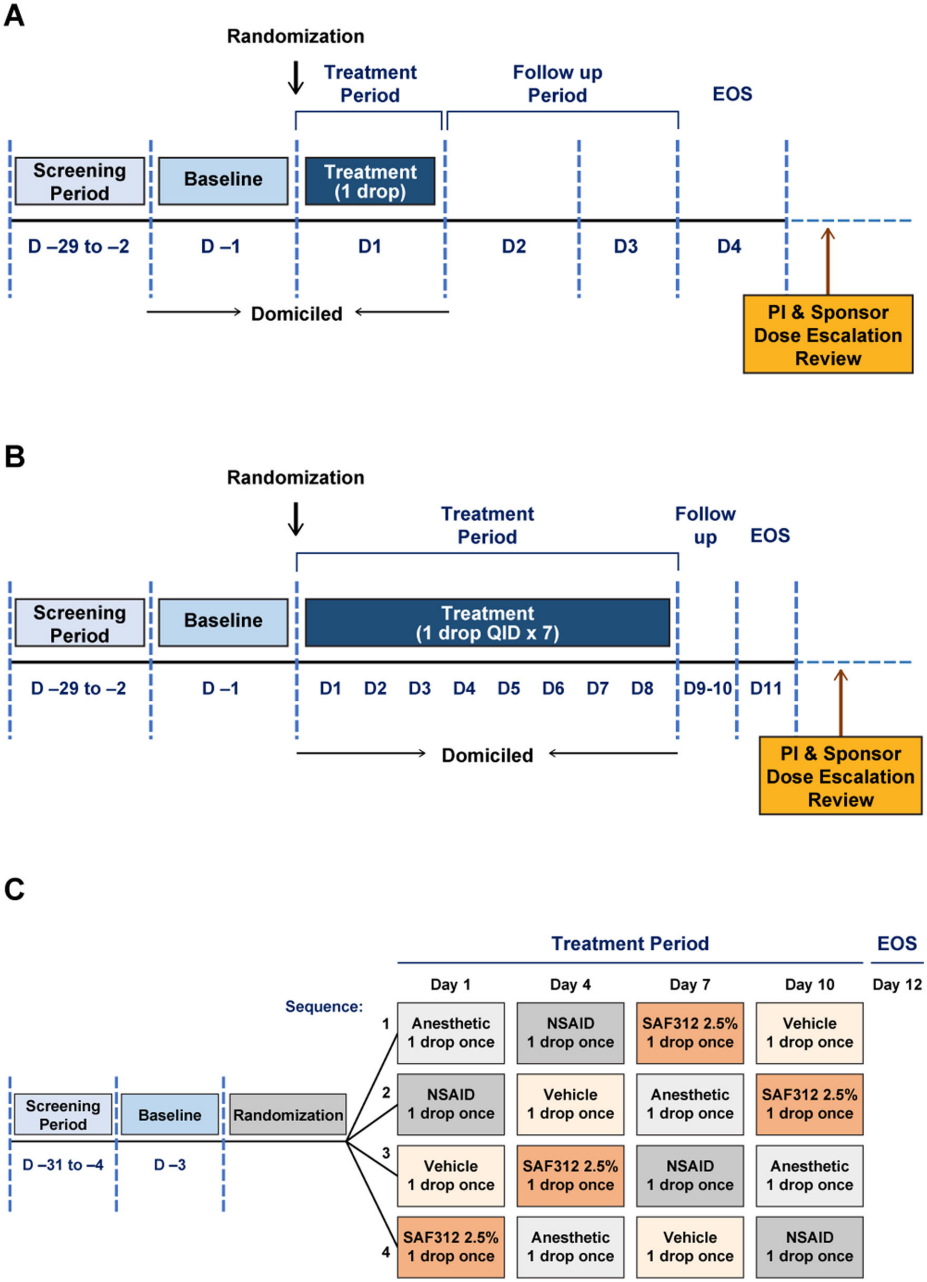
**Study design** **.** (**A**) SAD. (**B**) MAD. (**C**) Esthesiometry. D, day; EOS, end of study; MAD, multiple ascending dose; NSAID, non-steroidal anti-inflammatory drug; PI, principal investigator; SAD, single ascending dose; QID, 4 times daily.

This FIH study was a single-center, double-masked, randomized, vehicle-controlled study conducted between November 2015 and October 2016 in healthy participants. The study comprised three parts: a single-ascending dose (SAD), a multiple-ascending dose (MAD), and an esthesiometry part (Fig. 1). Each dose cohort (SAF312 0.15%, 1.5%, and 2.5%) of SAD and MAD parts included 8 healthy participants randomized 3:1 to receive either SAF312 or vehicle: 1 drop once (SAD) or 1 drop 4 times or 8 times (supratherapeutic dose at the maximum feasible concentration of 2.5%) daily for 7 days (MAD). The MAD part was conducted once the safety data of the SAD part was available and deemed satisfactory. Esthesiometry was commenced only after the SAF312 maximum feasible concentration of 2.5% 4 times daily was established as well-tolerated in cohort 3 of the MAD part. The esthesiometry part comprised the 1 dose cohort (SAF312 2.5%), 12 healthy participants were randomized equally to 4 sequences consisting of a single drop of SAF312 2.5%, tetracaine 0.5% (anesthetic), diclofenac sodium 0.1% (NSAID), or vehicle in a Williams square design.^32^

### SAF312 Formulation

The formulation is a white to off white, uniform, suspension with a pH of approximately 7.4. The formulation contains SAF312 along with the following standard pharmacopeia excipients: tyloxapol, carbopol, glycerin, sodium chloride, tromethamine, hydrochloric acid to adjust pH, and purified water.

### Study Subjects

Healthy male and female participants aged 18 to 50 years with normal medical history, physical examination, vital signs, electrocardiogram (ECG), and a body mass index of 18 to 29 kg/m^2^ were included. The main exclusion criteria were women of childbearing potential; subjects with a history of any ocular surgery or laser within the past 6 months; any chronic eye disease other than refractive error, incipient cataract, strabismic amblyopia, or anisometropic amblyopia; acute eye disease within the past 6 months; any currently active ocular condition; and subjects who were using contact lenses at the time of the study or had used them in the past 3 years.

The study was conducted in accordance with guidelines on good clinical practice and with ethical principles established by the Declaration of Helsinki. An institutional review board in the United States approved the study protocol, and each participant provided written informed consent.

### Objectives

The primary objectives of the study were to assess the safety (ocular and systemic) and tolerability of single and multiple doses of topical ocular SAF312 at various concentrations and dosing frequencies and explore the MTD. The secondary objective was to assess the systemic exposure after single and multiple doses of topical ocular SAF312 at various time points. The exploratory objectives were to explore the potential effect of topical ocular SAF312 on ocular hyperemia, tear production (Schirmer test without anesthesia), tear film break-up time (TFBUT), and blink rate in the MAD groups; anesthetic effect on the eye was assessed after topical ocular administration using esthesiometry.

### Assessments

#### Safety and Tolerability Assessments

Safety and tolerability were assessed by monitoring AEs, vital signs, and ECG, and performing clinical laboratory assessments and hand immersion test at 49°C (in the MAD part only). All recorded AEs were listed and tabulated by system organ class, preferred term in accordance with MedDRA (versions 18.0, 18.1, and 19.0). Vital signs, ECG, and clinical laboratory assessments were assessed at screening and throughout the study period. Hand immersion test was performed in a water bath at 49°C and the time to discomfort-induced withdrawal was recorded. The test was conducted at screening, baseline (BL), and postdose days 2, 5, 8, and 9 in the MAD part. Ocular safety assessments included Early Treatment Diabetic Retinopathy Study (ETDRS) best-corrected visual acuity (BCVA) score, intraocular pressure (IOP), slit lamp biomicroscopy, and fluorescein corneal staining (NEI scale 0–3) and were assessed at screening, BL, postdose days 1, 2, 3, and 4 in the SAD part, and at screening, BL, postdose days 1, 2, 5, 8, and 9 in the MAD part.

Ocular hyperemia was assessed at screening, BL, and postdose on days 1, 2, 3, and 4 in the SAD part and at screening, BL, and postdose on days 1, 2, 5, 8, and 9 in the MAD part using the McMonnies redness scale^33^ in 4 regions of the bulbar conjunctiva (superior, inferior, temporal, and nasal). Each eye and each region were graded by severity (0–5). Blink rate, tear production, and TFBUT were assessed at screening, BL, and postdose on days 1, 2, 5, 8, 9, and 10. Blink rate was evaluated as blinks per minute; tear production was assessed using a Schirmer test without anesthesia.^34^ TFBUT was assessed as the time until tiny dry spots develop in the tear film after fluorescein dye is added to the eye, under the slit lamp while the participant avoided blinking.

Corneal sensitivity was evaluated by esthesiometry testing, that is, measurement of the filament length (centimeters) at which cornea touch is perceived. For each participant, the average of the corneal sensitivity measured in both eyes at each time point was recorded for analysis (see Supplementary Section S1 and S2 for a detailed description). The overall summary statistics of the average corneal sensitivity at screening will be reported. Esthesiometry assessments were performed at screening and postdose on days 1, 4, 7, and 10.

#### PK Assessments

Blood samples were obtained from all participants at all studied dose concentrations in the SAD and MAD parts and evaluated postdose on days 1, 2, 3, and 4 in the SAD part, and postdose on days 2, 5, 6, 7, 8, and 9 in the MAD part. SAF312 was quantified using a validated liquid chromatography tandem mass spectrometry (LC-MS/MS) method; the lower limit of quantification (LLOQ) was 0.05 ng/mL. Concentrations were expressed in nanograms per milliliter. Plasma concentrations below the LLOQ were reported as “zero” and included in the calculation of summary statistics; missing data were labeled in the bioanalytical data report. PK parameters were determined with Phoenix WinNonlin (version 6.4) and included: maximum plasma concentration (C_max_), time to C_max_ (T_max_), area under the concentration-time curve from time of administration up to the time of the last quantifiable concentration (AUC_last_), AUC to infinity (AUC_inf_), AUC for a dosing interval (AUC_tau_), last measurable concentration (C_last_), minimum concentration (C_min_), time to C_last_ (T_last_), and accumulation rate (R_acc_), from the plasma concentration-time data.

### Statistical Analyses

All participants who received at least one dose of the study drug were included in the safety data set. There were no formal statistical hypotheses in this study. The sample size was determined by convention. With 6 participants exposed to active treatment in each cohort, there was at least an 80% probability of observing an AE with an incidence rate of 25%. Vital signs, clinical laboratory assessments, and ocular assessments were listed for each part, treatment, participant, and visit. Descriptive statistics were provided per treatment, cohort, and visit.

Ocular hyperemia, blink rate, tear production, and TFBUT were summarized by treatment and visit. For esthesiometry, Cochet-Bonnet esthesiometer was used. Corneal sensitivity measured by filament length (cm) were used as the dependent variable in a mixed model with repeated measures that is appropriate for William's square design. Pairwise treatment comparisons at all time points were made, and there were no adjustments for multiplicity. Summary statistics for plasma concentrations and parameter values included the mean (arithmetic and geometric), standard deviation (SD). Because T_max_ is generally evaluated by a nonparametric method, the median, minimum, and maximum were presented. Concentrations below the LLOQ were treated as zero in summary statistics. A geometric mean was not reported if the data set included zero values. The dose-concentration relationship was examined using a power model (PK = α dose^β^).

## Results

### Baseline Characteristics

In total, 24 participants were randomized in the SAD part of the study, of whom 23 completed the study (1 participant discontinued the study due to an AE [migraine] on day 1). In the MAD part, 32 participants were randomized and 31 completed the study (1 participant discontinued the study due to an AE [eye irritation] on day 6). All 12 participants enrolled in the esthesiometry part completed the study.

The demographic characteristics were overall balanced between SAF312- and vehicle-treated participants in the SAD and MAD parts and between the 4 treatment sequences in the esthesiometry part (Table 1).

**Table 1.** Summary of Demographic Data

		Gender n (%)	
	Age, y, Mean (SD)	Male	Female
**SAD**			
**SAF312 (0.15%), N = 6**	33.3 (4.08)	1 (16.7)	5 (83.3)
**SAF312 (1.5%), N = 6**	28.3 (5.96)	1 (16.7)	5 (83.3)
**SAF312 (2.5%), N = 6**	32.0 (11.45)	0 (0.0)	6 (100.0)
**Vehicle, N = 6**	37.7 (9.00)	1 (16.7)	5 (83.3)
**Total, N = 24**	32.8 (8.31)	3 (12.5)	21 (87.5)
**MAD**			
**SAF312 (0.15%)** **4 times daily, N = 6**	37.2 (5.95)	1 (16.7)	5 (83.3)
**SAF312 (1.5%)** **4 times daily, N = 6**	34.2 (7.52)	2 (33.3)	4 (66.7)
**SAF312 (2.5%)** **4 times daily, N = 6**	32.0 (4.24)	6 (100.0)	0 (0.0)
**SAF312 (2.5%)** **8 times daily, N = 6**	39.0 (9.51)	3 (50.0)	3 (50.0)
**Vehicle, N = 8**	35.3 (9.75)	2 (25.0)	6 (75.0)
**Total, N = 32**	35.5 (7.71)	14 (43.8)	18 (56.3)
**Esthesiometry**			
**Sequence 1, N = 3**	30.7 (13.61)	0 (0.0)	3 (100.0)
**Sequence 2, N = 3**	32.3 (10.21)	2 (66.7)	1 (33.3)
**Sequence 3, N = 3**	36.3 (9.02)	1 (33.3)	2 (66.7)
**Sequence 4, N = 3**	38.3 (14.22)	0 (0.0)	3 (100.0)
**Total, N = 12**	34.4 (10.70)	3 (25.0)	9 (75.0)

MAD, multiple ascending dose; N, number of participants enrolled; SAD, single ascending dose; SD, standard deviation; Sequence 1, tetracaine 0.5%/diclofenac 0.1%/SAF312 2.5%/vehicle; Sequence 2, diclofenac 0.1%/vehicle/tetracaine 0.5%/SAF312 2.5%; Sequence 3, vehicle/SAF312 2.5%/diclofenac 0.1%/tetracaine 0.5%; Sequence 4, SAF312 2.5%/tetracaine 0.5%/vehicle/diclofenac 0.1%.

### Safety and Tolerability Outcomes

#### Adverse Events

Overall, SAF312 demonstrated safety and tolerability in every dose tested, including single and multiple doses of 0.15%, 1.5%, and 2.5%, up to the maximum feasible concentration of 2.5% as single eye drop administration 8 times daily for 7 days. Therefore, MTD was not determined based on AEs; instead, the safe and well-tolerated supratherapeutic dose of SAF312 2.5% 8 times daily for 7 days was considered the MTD in this study as this was the highest dose tested.

In the SAD, MAD, and esthesiometry parts, there were no serious AEs; most of the reported AEs were mild in severity. No deaths or serious AEs were reported in this study.

In the SAD part, a total of 10 AEs (6 ocular and 4 non-ocular) were reported in 6 participants, of whom 3 received SAF312 (0.15% and 1.5%) and 3 received vehicle. No AE was observed in the SAF312 (2.5%) treatment cohort. Six ocular AEs (2 in SAF 0.15%, 3 in SAF 1.50%, and 1 in vehicle) were reported in 4 participants (all mild in severity); of these, 5 were suspected to be drug related. There were 4 non-ocular AEs (1 in SAF 0.15% and 3 in vehicle) reported in 3 participants (3 mild and 1 moderate) in the SAD part, and all were suspected to be drug related (Table 2). No dose-related increase in the number of AEs or severity was observed.

**Table 2.** Very Low Incidence of Only Mild AEs Across all Doses of Ocular SAF312 Eye Drops in SAD

SAD	SAF312 0.15% N = 6			SAF312 1.5% N = 6			SAF312 2.5% N = 6			Vehicle N = 6			Total N = 24
AEs by Preferred Term	n (%)	Drug Related	Severity	n (%)	Drug Related	Severity	n (%)	Drug Related	Severity	n (%)	Drug Related	Severity	n (%)
**Ocular AEs**													
Dry eye	0	–	–	1 (16.7)	Yes	Mild	0	–	–	0	–	–	1 (4.2)
Eye irritation	1 (16.7)	Yes	Mild	0	–	–	0	–	–	0	–	–	1 (4.2)
Eye pruritus	0	–	–	1 (16.7)	Yes	Mild	0	–	–	0	–	–	1 (4.2)
Ocular hyperemia	0	–	–	0	–	–	0	–	–	1 (16.7)	No	Mild	1 (4.2)
Scleral hyperemia	1 (16.7)	Yes	Mild	0	–	–	0	–	–	0	–	–	1 (4.2)
Feeling hot	0	–	–	1 (16.7)	Yes	Mild	0	–	–	0	–	–	1 (4.2)
**Non-ocular AEs**													
Headache	1 (16.7)	Yes	Mild	0	–	–	0	–	–	1 (16.7)	Yes	Mild	2 (8.3)
Dizziness	0	–	–	0	–	–	0	–	–	1 (16.7)	Yes	Mild	1 (4.2)
Migraine	0	–	–	0	–	–	0	–	–	1 (16.7)	Yes	Mod	1 (4.2)

AE, adverse event; Mod, moderate; N, number of participants enrolled and who received the study drug; n, number of participants with at least one AE in the category; SAD, single ascending dose.

In the MAD part, a total of 38 AEs (22 ocular and 16 non-ocular) were reported in 15 participants, of whom 12 received SAF312 (all dose concentrations) and 3 received vehicle. Twenty-two ocular AEs (4 in SAF 0.15%, 6 in SAF 1.50%, 6 in SAF 2.50% and 6 in vehicle) were reported (20 mild and 2 moderate); all were suspected to be drug related. There were 16 non-ocular AEs (2 in SAF 0.15%, 6 in SAF 1.50%, 1 in SAF 2.50%, and 7 in vehicle) reported (15 mild and 1 moderate) in the MAD part, of which 8 were suspected to be drug related (Table 3). No dose-related increase in number of AEs or severity was observed.

**Table 3.** Incidence of Ocular and Non-Ocular AEs was Low and Comparable With That of Vehicle Across the MAD Including the Supratherapeutic Dosing of SAF312 Eye Drops (8 Times Daily for 7 Days)

	SAF312 0.15% 4 Times Daily N = 6			SAF312 1.5% 4 Times Daily N = 6			SAF312 2.5% 4 Times Daily N = 6			SAF312 2.5%8 Times Daily N = 6			Vehicle N = 8			Total N = 32
MAD AEs by Preferred Term	n (%)	Drug Related	Severity	n (%)	Drug Related	Severity	n (%)	Drug Related	Severity	n (%)	Drug Related	Severity	n (%)	Drug Related	Severity	n (%)
**Ocular AEs**																
Anterior chamber flare	1 (16.7)	Yes	Mild	0	–	–	0	–	–	0	–	–	1 (12.5)	Yes	Mild	2 (6.3)
Anterior chamber inflammation	1 (16.7)	Yes	Mild	0	–	–	0	–	–	0	–	–	1 (12.5)	Yes	Mild	2 (6.3)
Corneal disorder^a^	1 (16.7)	Yes	Mild	0	–	–	0	–	–	0	–	–	1 (12.5)	Yes	Mild	2 (6.3)
Eye disorder^b^	1 (16.7)	Yes	Mild	0	–	–	0	–	–	0	–	–	1 (12.5)	Yes	Mild	2 (6.3)
Ocular hyperemia	0	–	–	0	–	–	1 (16.7)	Yes	Mild	1 (16.7)	Yes	Mild	0	–	–	2 (6.3)
Vision blurred^c^	0	–	–	2 (33.3)	Yes	Mild	0	–	–	0	–	–	0	–	–	2 (6.3)
Eye discharge	0	–	–	0	–	–	1 (16.7)	Yes	Mild	0	–	–	0	–	–	1 (3.1)
Eye irritation	0	–	–	0	–	–	1 (16.7)	Yes	Mod	0	–	–	0	–	–	1 (3.1)
Eye pruritus	0	–	–	1 (16.7)	Yes	Mod	0	–	–	0	–	–	0	–	–	1 (3.1)
Vital dye staining cornea present^d^	0	–	–	1 (16.7)	Yes	Mild	1 (16.7)	Yes	Mild	0	–	–	1 (12.5)	Yes	Mild	3 (9.4)
**Non-ocular AEs**																
Headache	1 (16.7)	Yes	Mild	1 (16.7)	Yes	Mild	0	–	–	0	–	–	0	–	–	2 (6.3)
Paresthesia	0	–	–	1 (16.7)	Yes	Mild	0	–	–	0	–	–	1 (12.5)	No	Mild	2 (6.3)
Gilbert's syndrome	0	–	–	1 (16.7)	No	Mild	0	–	–	0	–	–	0	–	–	1 (3.1)
Constipation	0	–	–	1 (16.7)	No	Mild	0	–	–	0	–	–	0	–	–	1 (3.1)
Flatulence	0	–	–	1 (16.7)	No	Mild	0	–	–	0	–	–	0	–	–	1 (3.1)
Hypertriglyceridemia	0	–	–	0	–	–	0	–	–	0	–	–	1 (12.5)	Yes	Mild	1 (3.1)
Pregnancy	1 (16.7)	No	Mild	0	–	–	0	–	–	0	–	–	0	–	–	1 (3.1)
Insomnia	0	–	–	0	–	–	0	–	–	0	–	–	1 (12.5)	Yes	Mild	1 (3.1)
Dysuria	0	–	–	0	–	–	0	–	–	1 (16.7)	No	Mild	0	–	–	1 (3.1)
Urine odor abnormal	0	–	–	1 (16.7)	Yes	Mild	0	–	–	0	–	–	0	–	–	1 (3.1)
Cough	0	–	–	0	–	–	0	–	–	0	–	–	1 (12.5)	No	Mild	1 (3.1)
Dry throat	0	–	–	0	–	–	0	–	–	0	–	–	1 (12.5)	No	Mild	1 (3.1)
Dyspnea	0	–	–	0	–	–	0	–	–	0	–	–	1 (12.5)	Yes	Mod	1 (3.1)
Pruritus generalized	0	–	–	0	–	–	0	–	–	0	–	–	1 (12.5)	Yes	Mild	1 (3.1)

AE, adverse event; N, number of participants enrolled and who received the study drug; n, number of participants with at least 1 AE in the category; MAD, multiple ascending doses; Mod, moderate.

a “Active inflammation or structural change in the cornea” was reported in 2 participants.

b “Inflammation, structural change or discharge, eyelids/conjunctiva” was reported in 2 participants.

c “Blurred vision in the left and right eyes respectively” was reported in 2 participants.

d “Vital dye staining cornea” was reported in 3 participants.

In the esthesiometry part, 5 AEs of mild severity were reported in 5 participants: 2 each in the diclofenac and SAF312 groups and 1 in the vehicle group. Of these AEs all but one (in diclofenac) were suspected to be study drug related.

Two participants discontinued the study drug due to AEs; both events were suspected to be related to the study drug (1 participant treated with vehicle in the SAD part had an AE of migraine on day 1, and 1 participant in the SAF312 2.5% 4 times daily in the MAD part had an AE of eye irritation on day 6).

#### Systemic and Ocular Safety Outcomes

No clinically significant changes were observed in vital signs, ECG, laboratory parameters, or hand immersion test. The hand immersion test (conducted only in the MAD part) showed no impact on heat sensitivity, consistent with the observed minimal systemic exposure. All participants in the treatment cohorts withdrew their hand from the water at 49°C at a time less than 22 seconds, with no difference between SAF312 and vehicle-treated participants. No clinically significant changes were observed in ocular assessments of BCVA score, IOP, slit lamp biomicroscopy, corneal staining, and dilated eye examination after administration of all the topical ocular SAF312 doses tested.

### Exploratory Ocular Outcomes and Corneal Sensitivity

#### Ocular Hyperemia

Ocular hyperemia, of grade 2 mild severity, was reported in 1 participant in the SAD part (1 participant treated with vehicle had ocular hyperemia in both eyes on day 1; the event resolved by day 3 and was not suspected to be study drug related) and in 2 participants in the MAD part (1 participant in SAF312 2.5% 4 times daily group experienced ocular hyperemia along with eye discharge and eye irritation on day 6 of the study in the left eye, and the study drug was permanently discontinued due to eye irritation, ocular hyperemia resolved on the same day of study drug discontinuation, and eye irritation resolved by day 8; 1 participant treated with SAF312 2.5% 8 times daily had ocular hyperemia on day 9 in the right eye, the event resolved by day 11 and no action was taken on the study drug). Both the events in the SAF312 groups in the MAD part were suspected to be related to the study drug by the investigators. In all the remaining participants, in the SAD and MAD parts, the redness score was either grade 0 or grade 1 on McMonnies redness scale.

#### Blink Rate, Tear Production, and TFBUT

No clinically relevant changes were observed for blink rate, tear production, and TFBUT (Supplementary Figures S3, S4, S5) with the SAF312 eye drops in the MAD part. These assessments were conducted only in the MAD part.

#### Corneal Sensitivity by Esthesiometry

**Figure 2.**
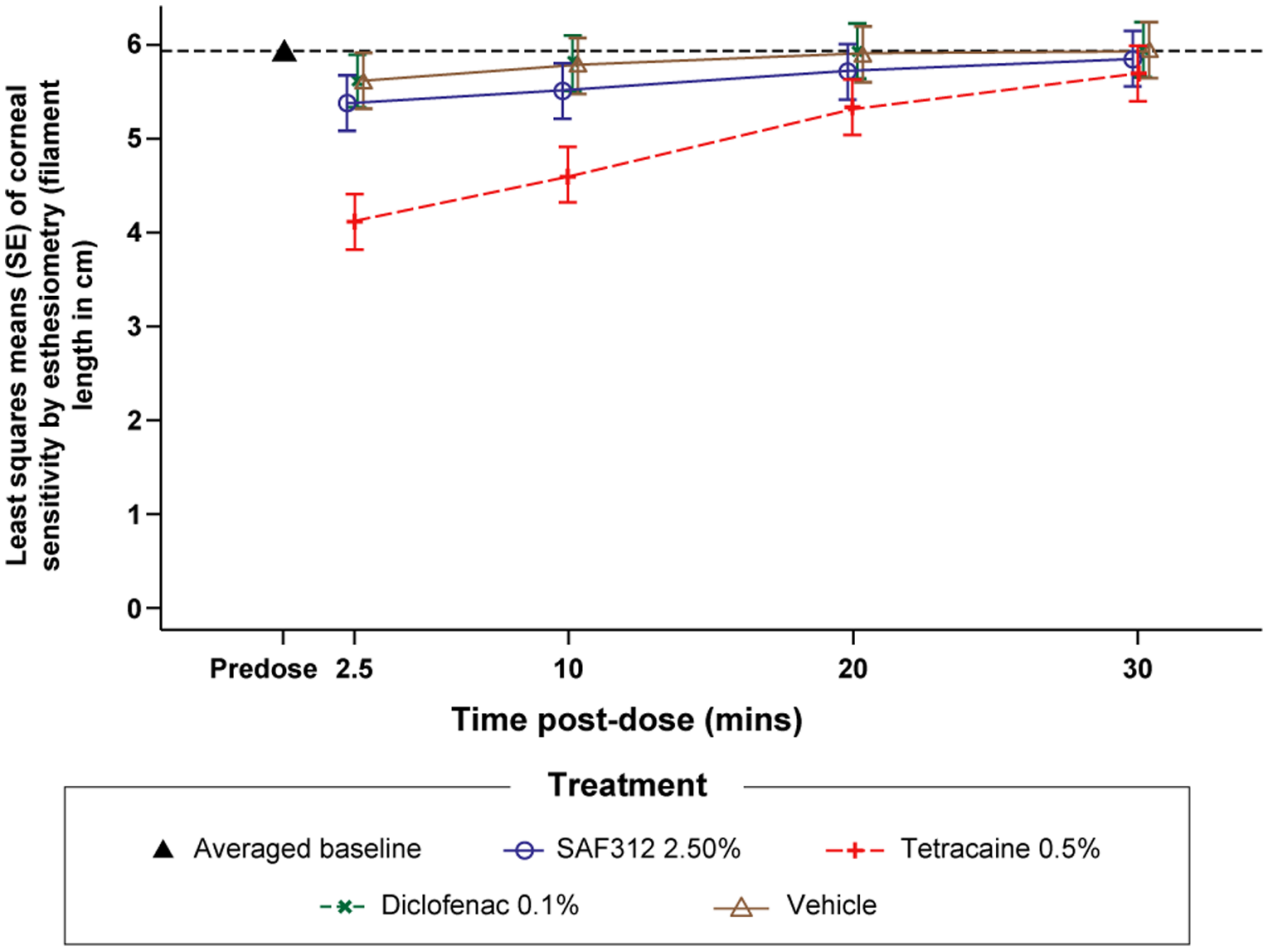
Least square means (SE) from the statistical analysis of corneal sensitivity by esthesiometry (filament length in cm; study eye; Esthesiometry). SE, standard error.

No difference was seen in corneal sensitivity between SAF312 2.5% and vehicle (negative control), with all *P* values ≥ 0.395 at scheduled time points up to 30 minutes postdose, whereas the mean values of SAF312 2.5% were numerically slightly lower than those of vehicle. Corneal sensitivity after tetracaine 0.5% (anesthetic, as positive control) was decreased compared with vehicle at 2.5 and 10 minutes postdose, with *P* < 0.001 for both time points. Corneal sensitivity after SAF312 2.5% was higher than tetracaine 0.5% at 2.5 and 10 minutes after dosing, with *P* < 0.001 and *P* = 0.007, respectively. SAF312 showed no anesthetic effect on the cornea, similar to vehicle (negative control) and diclofenac (NSAID), as opposed to the anesthetic eyedrop tetracaine (Fig. 2).

### Pharmacokinetics

**Figure 3.**
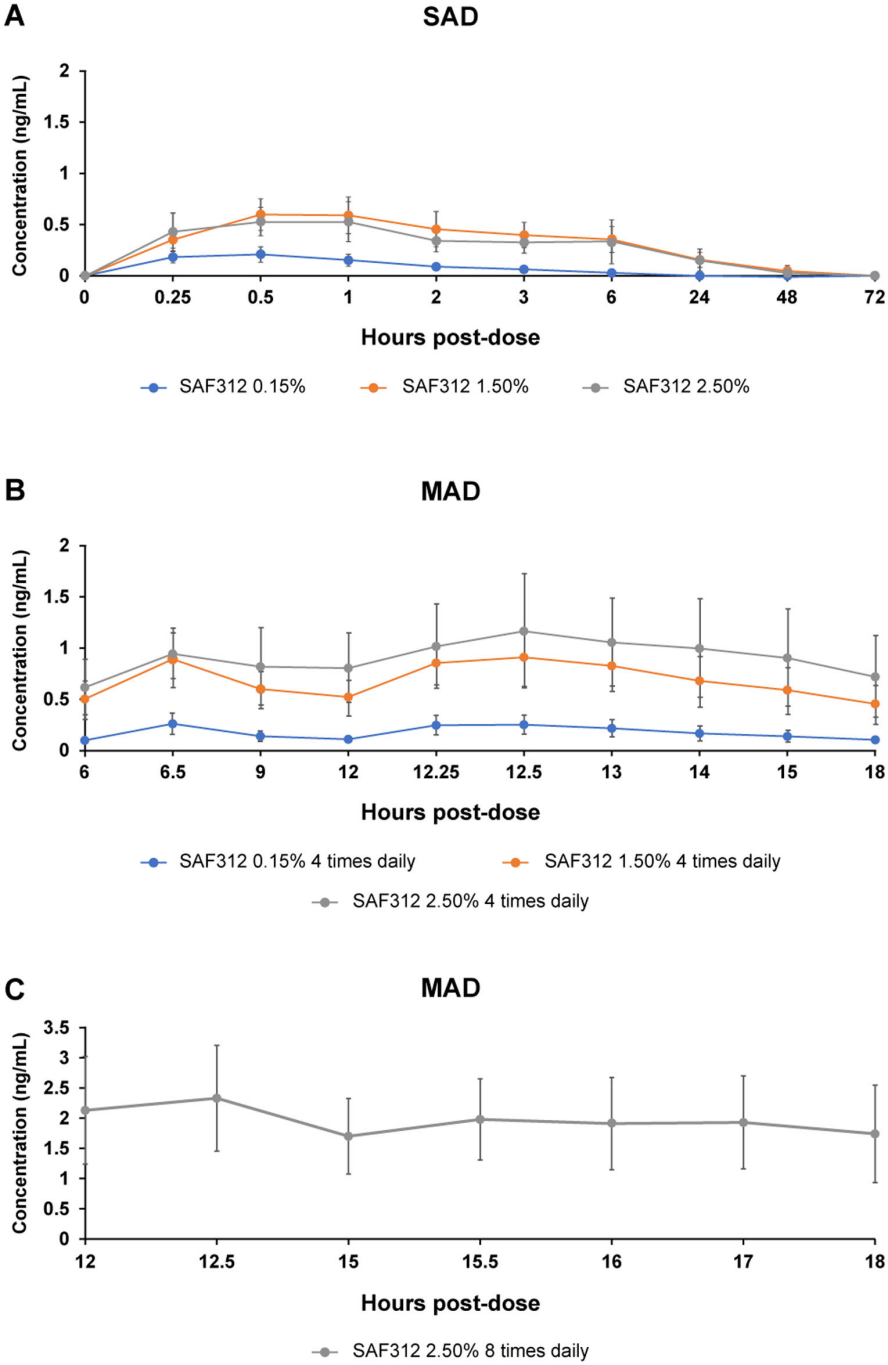
Plasma concentration time profiles of SAF312. (**A**) Arithmetic mean (SD) plasma concentration-time profile of SAF312 after administration of a single eye drop of 0.15% (0.055 mg), 1.5% (0.555 mg), or 2.5% (0.925 mg) suspension dose in healthy participants. (**B**) Arithmetic mean (SD) plasma concentration-time profile of SAF312 from the third dose on day 8 with 4 times daily administration of a single eye drop of 0.15% (0.055 mg), 1.5% (0.555 mg), or 2.5% (0.925 mg) suspension dose in healthy participants. (**C**) Arithmetic mean (SD) plasma concentration-time profile of SAF312 from sixth dose on day 8 with 8 times daily administration of a single eye drop of 2.5% (0.925 mg) suspension dose in healthy participants. MAD, multiple ascending dose; SAD, single ascending dose; SD, standard deviation.

SAF312 was rapidly absorbed into the systemic circulation with a median T_max_ of approximately 0.25 to 0.75 hours after single dose or after multiple doses on days 2 to 8. The systemic exposure was low following both single and multiple doses with moderate variability across all doses. After single dose administration, the highest mean C_max_ of 0.631 ng/mL and mean AUC_last_ of 9.08 h × ng/mL were observed with SAF312 1.5% strength. Upon multiple dose administration, the highest arithmetic mean C_max_ of 2.03 ng/mL, mean AUC_tau_ value of 5.64 h × ng/mL (tau = 3 hours), and calculated AUC_0–24h_ (AUC_tau_, 5.64 hours × ng/mL × 8) of 45.1 hours × ng/mL were observed with 8 times daily dosing of SAF312 2.5% on day 8 (Table 4). Increasing doses from 0.15% to 2.5% resulted in a less-than-dose-proportional increase in systemic exposure. Increase in SAF312 exposure from day 2 to 8 at steady-state was minor (1.7-fold increase) following administration of SAF312 0.15%, 1.5%, or 2.5% suspension 4 times daily and following 2.5% 8 times daily for 7 days (1.3-fold increase; Fig. 3).

**Table 4.** Summary PK Statistics of SAF312 Plasma Parameters Following Single or Multiple Doses

Dose	Profile Day	Statistic	C_max_ (ng/mL)	T_max_ (h)#	AUC_last_ (h × ng/mL)	C_last_ (ng/mL)	T_last_ (h)#	
**SAD**								
0.15%	1	n mean (SD)	6 0.212 (0.0751)	6 0.500 [0.250; 0.517]	6 0.466 (0.236)	6 0.0577 (0.00406)	6 4.50 [2.00; 6.00]	
1.5%	1	n mean (SD)	6 0.631 (0.189)	6 0.750 [0.500; 1.00]	6 9.08 (4.45)	6 0.0907 (0.0143)	6 36.0 [24.0; 48.2]	
2.5%	1	n mean (SD)	6 0.556 (0.177)	6 0.500 [0.500; 1.00]	6 7.54 (6.00)	6 0.112 (0.0256)	6 24.0 [24.0; 48.0]	
					AUC_last_	AUC_tau_^3^	AUC_0–24_^2^	
Dose	Profile Day	Statistic	C_max_ (ng/mL)	T_max_^1^ (h)	(h × ng/mL)	(h × ng/mL)	(h × ng/mL)	R_acc_^4^

**MAD**								
0.15% 4 times daily	2	n mean (SD)	6 0.222 (0.0692)	6 0.250 [0.233; 0.500]	6 0.714 (0.310)	6 0.725 (0.304)		
	8	n mean (SD)	6 0.272 (0.0929)	6 0.375 [0.250; 0.500]	6 1.02 (0.356)	6 1.02 (0.356)	6 4.10 (1.42)	6 1.66 (0.840)
1.5% 4 times daily	2	n mean (SD)	6 0.797 (0.406)	6 0.500 [0.250; 1.00]	6 3.27 (1.78)	6 3.30 (1.80)		
	8	n mean (SD)	6 0.941 (0.252)	6 0.375 [0.250; 0.500]	6 3.77 (1.33)	6 3.77 (1.33)	6 15.1 (5.31)	6 1.35 (0.508)
2.5% 4 times daily	2	n mean (SD)	6 0.873 (0.226)	6 0.500 [0.500; 1.00]	6 3.65 (0.902)	6 3.69 (0.912)		
	8	n mean (SD)	5 1.21 (0.533)	5 0.517 [0.250; 1.00]	5 5.55 (2.69)	5 5.55 (2.69)	5 22.2 (10.8)	5 1.62 (0.817)
2.5% 8 times daily	2	n mean (SD)	6 1.48 (0.385)	6 0.517 [0.500; 2.00]	6 3.77 (1.11)	4 3.27 (0.527)		
	8	n mean (SD)	6 2.03 (0.752)	6 0.750 [0.500; 3.00]	6 5.64 (2.22)	6 5.64 (2.22)	6 45.1 (17.8)	4 1.31 (0.0603)

#For T_max_ and T_last_, median [range] are reported instead of mean (SD).

AUC, area under the curve; h, hours; MAD, multiple ascending doses; n, number of patients; PK, pharmacokinetic; SAD, single ascending dose; SD, standard deviation; T_max_, time to maximum plasma concentration.

PK parameters for MAD are calculated from the third dose on days 2 and 8.

PK parameters for the supratherapeutic cohort are calculated from the sixth dose on days 2 and 8.

1 For T_max_, median value and range are reported.

2 AUC_tau_ multiplied by 4 (MAD) or by 8 (supratherapeutic cohort: 2.5% 8 times daily).

3 Tau is 0 to 6 hours for 4 times daily dosing in MAD and 0 to 3 hours for 8 times daily dosing schedule in the supratherapeutic cohort.

4 R_acc_ is ratio of AUC_tau_ on day 8 and AUC_tau_ on day 2.

## Discussion

The results from this FIH study demonstrated that SAF312 (Libvatrep) eye drops were generally safe and well-tolerated, with no dose related increase in number of AEs or severity observed, even with the maximum feasible concentration of 2.5% administered at a supratherapeutic dose (7.4 mg/d) of 1 drop 8 times daily (every 3 hours) in 1 eye for 7 days. After both single and multiple doses of topical SAF312 0.15%, 1.5%, and 2.5%, the plasma PK profile showed rapid topical absorption of SAF312 and low systemic exposure.

The expected dose frequency in clinical practice is up to 4 times daily, and any dosing more frequent than this can be considered “supratherapeutic.” In the current study, a dosing frequency of 8 times daily was selected as the supratherapeutic dosing to assess the safety and tolerability of SAF312. Because 2.5% was the maximum feasible concentration afforded with the current formulation, the highest dose tested in this study was the supratherapeutic dose of SAF312 2.5% 8 times daily for 7 days.

Topical ocular administration of SAF312, a selective TRPV1 inhibitor, showed no systemic safety concerns, such as impaired thermal perception, as demonstrated by no meaningful change in immersion time between SAF312 and vehicle-treated participants in the hand immersion test and mean body temperature comparable with that at BL. Furthermore, topical SAF312 has a much safer profile than oral TRPV1 inhibitors, which have typical AEs of heat insensitivity and transient increase in body temperature.^8^^,^^28^^,^^35^ This was consistent with much lower systemic exposure after topical ocular versus oral administration of SAF312. A low systemic exposure was maintained after topical ocular administration, even at the supratherapeutic dose (2.5% SAF312 1 drop 8 times daily in 1 eye for 7 days).

Most AEs observed were mild and similar between SAF312- and vehicle-treated healthy participants; no severe AEs, serious AEs, or deaths were reported. No clinically significant changes between screening and final examination were observed in laboratory and vital parameters or comprehensive ocular assessments.

The well-tolerated systemic safety profile of SAF312 could be attributed to the low systemic exposure after topical ocular administration. Furthermore, increasing the dose strength from 0.15% to 2.5% did not result in a dose-proportional increase in systemic exposure. Accumulation of SAF312 at steady-state was small (approximately 1.7-fold) following administration of a 0.15%, 1.5%, or 2.5% suspension 4 times daily and 2.5% 8 times daily for 7 days (approximately 1.3-fold). Overall, PK findings from this study suggest a minimal exposure risk even after 7 days of treatment at a 2.5% dose administered 8 times daily.

Corneal sensitivity is important for the maintenance of a healthy corneal epithelium and to protect a patient from potential harm.^36^ Repeated use of agents with an anesthetic effect can lead to decreased corneal sensations, frequently associated with persistent corneal and ocular surface damage, or accidental injury due to lack of sensation.^13^^–^^16^ Esthesiometry is typically used to assess corneal sensitivity in different pathologies, such as DED, and diabetic and herpetic keratitis, and to assess the recovery of corneal sensitivity after different types of refractive surgery. Topical dosing with SAF312 2.5% showed no anesthetic effect on the cornea unlike dosing with tetracaine, a frequently used corneal anesthetic eyedrop. Ocular anesthetics, such as tetracaine, have an established analgesic effect and are contraindicated for OSP due to their negative effect on wound healing.^13^^–^^16^ Preclinical findings in a rabbit PRK model demonstrated that topical ocular administration of SAF312 had no effect on corneal wound healing (Stasi K., et al. IOVS 2021;62: ARVO E-Abstract 3538424).

SAF312 eye drops use showed minimal to no effect on exploratory outcomes comprising the ocular surface health parameters, such as eye blink frequency, tear production without anesthesia, ocular hyperemia, and TFBUT. Continuous topical use of local anesthetics can increase the incidence of infection and corneal scarring, as well as impair the blink reflex.^37^ The absence of changes to these measures with SAF312 supports its tolerability and amenability in patients with OSP associated with ocular surface disease.^38^^–^^41^ Corneal staining is another commonly used diagnostic method to assess the ocular surface damage in various ocular conditions, including DED.^42^ No clinically relevant changes were observed in corneal staining after administration of topical ocular SAF312. The other ocular safety evaluations, such as ETDRS BCVA score, IOP, slit lamp biomicroscopy, and dilated eye examination, also showed no relevant changes with administration of topical SAF312 in healthy participants. Taken together, these observations demonstrate a well-tolerated ocular safety profile for topical SAF312.

Limitations of this study include the short treatment period and small sample size. Moreover, although SAF312 was not associated with an anesthetic effect at the concentrations tested in a single administration, its analgesic properties were not assessed. Future trials will provide more data on the safety and efficacy of topical ocular SAF312 for the management of OSP.

In conclusion, early safety findings from an FIH trial evaluating the topical ocular formulation of SAF312 suggest that SAF312 was well-tolerated, with no ocular or systemic safety concerns, observed up to a supratherapeutic dose (8 times daily for 7 days) of the maximum feasible concentration (2.5%) in healthy participants. SAF312 had no anesthetic effect on the cornea and demonstrated a rapid topical ocular absorption with low systemic exposure in healthy participants. These FIH results support further clinical development of topical ocular SAF312 as a potential treatment for alleviating OSP.

## Supplementary Material

Supplement 1
