# Supplementary material for: Topical Ocular TRPV1 Antagonist SAF312 (Libvatrep) Demonstrates Safety, Low Systemic Exposure, and No Anesthetic Effect in Healthy Participants
Source: Transl Vis Sci Technol. 2022 Nov 17;11(11):15. doi: 10.1167/tvst.11.11.15 (PMC9684620; doi:10.1167/tvst.11.11.15)
Supplement: Supplement 1 [file tvst-11-11-15_s001.pdf]

Supplementary material

S1: Esthesiometry Test

1. Holding the esthesiometer in your dominant hand (in the same way you would hold a pen), extend the length of the filament to 6.0 cm.
2. Select the eye to be tested. At Screen and Baseline visits, both eyes will be tested. On dosing days 1, 4, 7, and 10, only the dosed study eye will be tested.
3. Refer to the diagram in S2 for a schematic of the test.
4. If this is a Screening visit, start the examination with a non-contact test to evaluate the subject's reliability. If this is a not a Screening visit, skip the non-contact test.
5. **Non-contact test**: With the filament perpendicular to the surface of the cornea, approach the subject's eye. Just before the filament makes contact with the corneal center, move it away from the eye and ask if the subject felt anything. Repeat this procedure again (up-to three times).

   If the subject answers 'Yes' to two of three non-contact tests, this subject is not reliable, the test should be stopped, and the subject should be reported as a screen failure.

   If the subject says 'No' to two of three non-contact tests, continue with the testing.
6. With the filament extended to 6.0 cm and held perpendicular to the surface of the cornea, approach the subject's eye and very gently touch the filament to the central cornea, applying just enough pressure to see the filament bend. Carefully move the filament away from the eye and ask if the subject felt any contact to the eye. You may notice the subject flinch, however please ask the subject to respond verbally. Repeat up-to two more times with the same filament length.

7. If the subject does not feel the contact in 2 out of 3 tests, reduce the filament length in increments of 1.0 cm, performing three tests at each length, until the subject has responded 'Yes' to two of three tests with the same filament length.
8. Once two out of three affirmative responses are observed, increase the filament length by 0.5 cm and repeat.
9. Record **the longest filament length in cm** to which the subject responds 'Yes' two out of three times. For Screening visit, **if the recorded length for either eye is NOT between 5.0 and 6.0 cm (inclusive), this subject should be screen failed.**
10. Once the result has been recorded, retract the filament fully. Either continue on to the subject's second eye (if Screen or Baseline visit) or set aside the esthesiometer, and continue to the next subject using the appropriate esthesiometer.
11. Once all subjects have completed testing for the day, disinfect the filament tips and cone tips. Sterilize the cone tips and the filament tips if the esthesiometer will be used on a new subject or if accidental contact with anything other than the subject's eye.

S2: Diagram of esthesiometry test

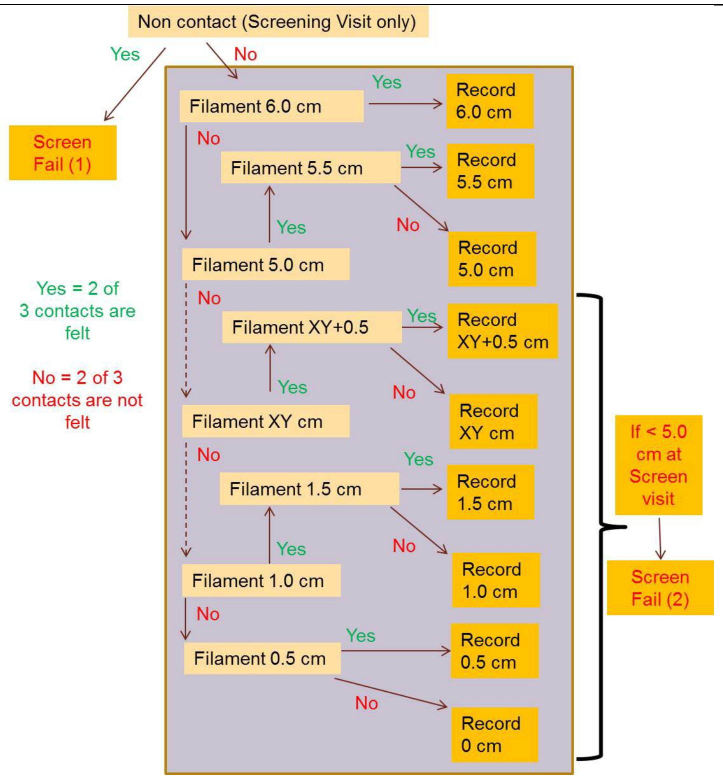

S3: Mean (SD) plot for blink rate (Part 2: MAD)

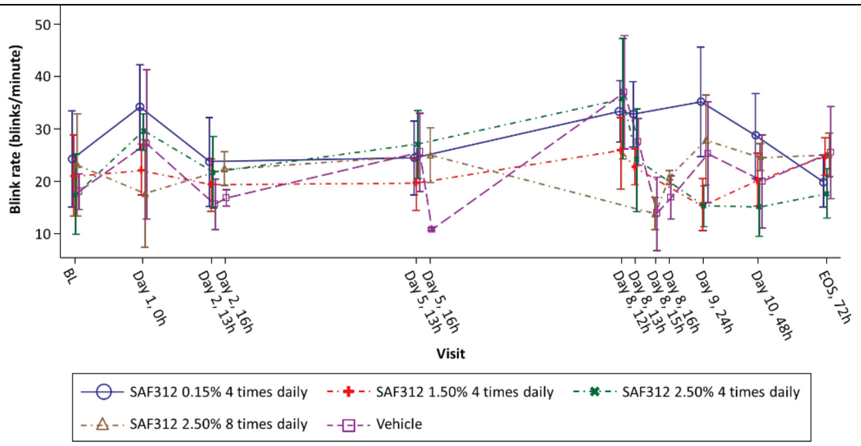

Vehicle subjects are pooled across all MAD cohorts (4 and 8 times daily dosing)

BL, baseline; MAD, multipe ascending dose; SD, standard deviation.

S4: Mean (SD) plot for tear production (study eye) (Part 2: MAD)

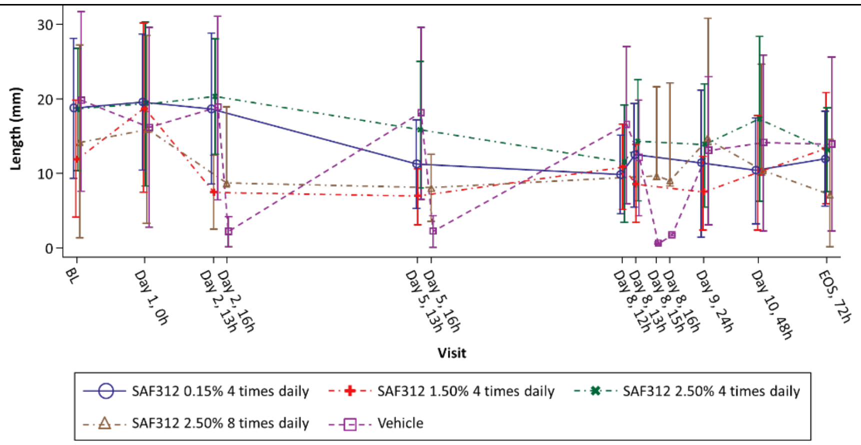

Vehicle subjects are pooled across all MAD cohorts (4 and 8 times daily dosing)

BL, baseline; MAD, multipe ascending dose; SD, standard deviation.

S5: Mean (SD) plot for TFBUT (study eye) (Part 2: MAD)

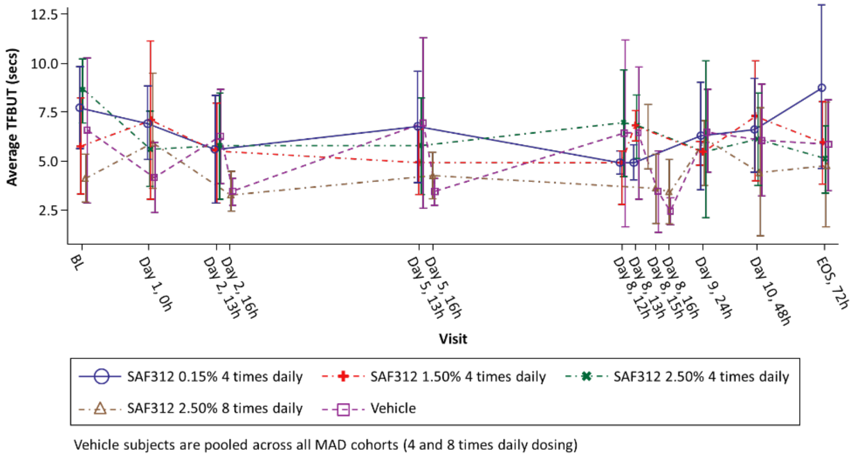

BL, baseline; MAD, multipe ascending dose; SD, standard deviation.
